# A Comparative Study on the Shaping Ability of K3, Profile and Protaper Instruments in Simulated Curved Root Canals

**Published:** 2010-08-15

**Authors:** Chinni Suneelkumar, Chellaswamy Savarimalai Karumaran, Sundararaman Ramachandran, Rajamani Indira, Padmabhan Shankar, Anil Kumar

**Affiliations:** 1 *Department of Endodontics, Department of Conservative Dentistry and Endodontics, Narayana Dental College and Hospital, India*; 2 *Department of Endodontics, Department of Conservative Dentistry and Endodontics, Ragas Dental College and Hospital, India*

**Keywords:** Endodontic Handpieces, Nickel-Titanium, Resin Blocks, Root Canal, Root Canal Shaping

## Abstract

**INTRODUCTION:** The objective of this study was to compare the shaping ability of three rotary filing systems; constant taper K3 instruments, constant taper ProFile instruments and progressive taper ProTaper rotary instruments in clear resin blocks with simulated curved root canals.

**MATERIALS AND METHODS:** Forty five resin blocks were divided into three groups. Group A preparation was conducted with K3, Group B with ProFile and Group C with ProTaper instruments. Pre and post instrumentation images were superimposed and assessment of the canal shape was completed with a computer image analysis program at 14 levels of the root canal system.

**RESULTS:** Group A inner and outer curvature pre and post instrumentation values were significantly different (P<0.05) at levels 3; at level 13 only the outer curvature and levels 6, 7, 8 the inner curvature had significantly different values between pre and post instrumentation. Group C had significant P values (P<0.05) at levels 2, 3, 4, 12, 13 in the outer curvature and at levels 6, 7, 8 of the inner curvature.

**CONCLUSION:** Overall, all three rotary instruments maintained root canal curvatures well. ProTaper instruments significantly removed more resin material from outer canal curvature in the apical third when compared to the other two groups.

## INTRODUCTION

Cleaning and shaping of the root canal space is a primary objective of root canal treatment (1). The shaping of the root canal system begins either from the coronal or from the apical parts of the root canal. The early preparation of the coronal part of the canal system is considered to be superior. Coronal preparations provides advantages like straighter access to the apical region, elimination of the dentinal interferences found in the coronal portion, and allows apical instrumentation to be accomplished quickly and efficiently (2).

The introduction of various Nickel-Titanium (NiTi) rotary instruments have several advantages over stainless steel instruments such as maintaining canal shape, decreasing the time taken for canal preparation, reducing operator and patient fatigue and the incidence of procedural error (3-5).

K3 (SybronEndo, West Collins, CA) has an asymmetrical constant tapered active file design, slight positive rake angle, with variable helical flute angle and a variable core diameter, which allow improved debris removal and a cutting rather than a planning action (6).

ProFile (Dentsply, Maillefer, Ballaigues, Switzerland) instruments are made by machining three equally spaced U-shaped grooves around the shaft of a taper NiTi wire. There is a central parallel core inside that may account for the enhanced flexibility. ProFile file has a bullet-nosed tip with a rounded transition angle (7).

ProTaper (Dentsply, Maillefer, Ballaigues, Switzerland) is an active file design, with a convex triangular cross-sectional design and an advanced flute design that combines multiple taper within the shaft (8).

**Figure 1:**
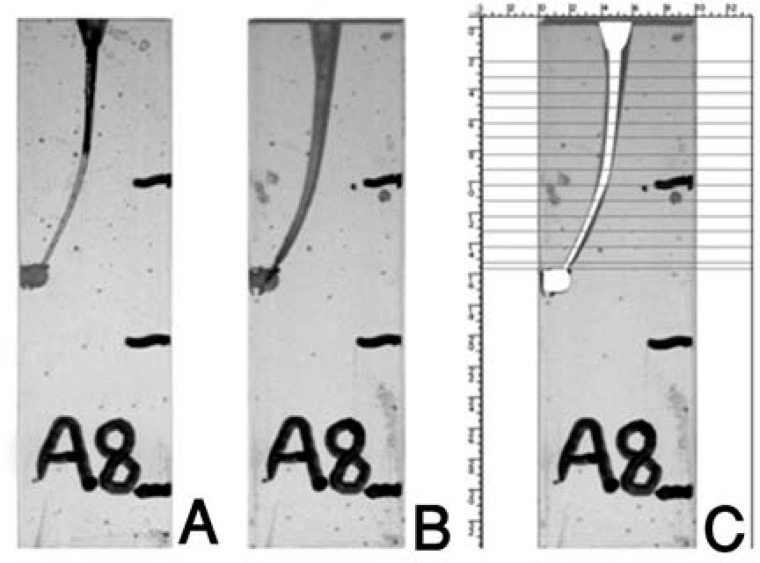
Resin Blocks A) Pre Instrumentation B) Post Instrumentation C) Super Imposition

The purpose of this *in vitro *study was to compare the shaping ability of constant taper K3 instrument with constant taper ProFile instrument as well as the progressive taper ProTaper instrument using prefabricated clear resin blocks with 35 degree root curvature simulated root canals (9).

## MATERIALS AND METHODS

***Simulated canals:***

Forty five simulated canals with 35 degree curvature made of clear polyester resin (SybronEndo, West Collins, CA, USA) were utilized. The Schneider method was employed for measuring the degree of curvature. The diameter was equivalent to an ISO standard size 15 instrument. Each block was 30 mm in height. The canals were 16.5 mm in length. The first 8 mm consisted of the coronal straight portion and the final 8.5 mm the curved portion. The radius of the curvature was 4 mm. The 45 blocks were equally divided into 3 groups of 15 each; *i.e. *group A (K3), B (ProFile), and C (ProTaper). Three markings were made with black marker from the superior aspect of each block at 10 mm intervals as reference points for future imaging.

The assessment of preparation shape was carried out with the computer image analysis program (Adobe Photoshop 7.0 Adobe systems Inc., San Jose, CA, USA). Pre-instrumentation images were taken using a digital camera (Minolta 5.2 megapixels, Japan) at a standard object-camera distance of 21 cm, and were then stored as a JPEG file into a computer. The canals were visualized at ×10 magnification and lines were drawn over the image at 1 mm intervals starting at 0.5 mm from the canal terminus up to 14.5 mm, using Adobe photoshop 7.0 software.

***Preparation of the simulated canals:***

The instrumentation was performed by a single operator with the help of X-smart rotary handpiece (X mart, Dentsply, Malliefer, Ballaigues, Switzerland)**.** Canals in all the three groups were prepared according to the manufacturers’ instructions. All three groups followed crown down technique at 300 RPM The torque was variable depending on the size and taper of the instrument. In group A, K3 body shapers were used up to the first 8 mm, K3 size 40 (all files had 6% taper) prepared the canal up to 10 mm, K3 size 35 were used up to 12mm, K3 size 30 file prepared the canal to 14mm and K3 size 25 was used for the remainder 16 mm. In Group B, ProFile orifice shapers prepared the canal for the first 8 mm, then ProFile size 40 (all files had 6% taper) were used up to 10 mm, ProFile size 35 were used up to 12 mm, ProFile size 30 were used till 14 mm and finally ProFile size 25 prepared the canal for the final 16 mm. In Group C, ProTaper Sx files were used for the first 8 mm, ProTaper S1 files were used till 12 mm and then ISO file size no. 15 was inserted to recapitulate to the working length. Subsequently, the canal was prepared with ProTaper S1 files and then ProTaper S2 files for the remaining 16 mm. Finally, ProTaper F1 files and then ProTaper F2 files were used for the 16 mm of canal length. The last apical file used was size 25 with 0.06 taper file in Group A and B and F2 in Group C in order to produce similar apical size enlargements in all the groups.

Canals were copiously irrigated with saline after every file and chelating agent Glyde File Prep (Dentsply Maillefer, Ballaigues, Switzerland) was applied as a lubricant.

Smaller size 15 and 20 K-files were used to remove debris and canals were dried with paper points.

**Table 1:** *Group A:* Canal enlargement at various levels with K3 instruments *(mm)*

**Levels**	**Outer Curvature **		**Inner Curvature**		**P value**
	**Mean**	**SD**	**Mean**	**SD**	
1	0.11	0.06	0.06	0.05	0.17
2	0.15	0.11	0.08	0.05	0.08
3	0.19	0.08	0.11	0.04	0.04 a
4	0.21	0.08	0.12	0.07	0.11
5	0.16	0.05	0.13	0.05	0.19
6	0.10	0.05	0.16	0.05	0.04 a
7	0.09	0.04	0.21	0.08	0.005 a
8	0.11	0.04	0.22	0.07	0.002 a
9	0.15	0.05	0.19	0.04	0.19
10	0.19	0.06	0.20	0.05	0.59
11	0.24	0.07	0.20	0.05	0.28
12	0.26	0.11	0.20	0.09	0.21
13	0.29	0.08	0.26	0.08	0.007 a
14	0.31	0.21	0.20	0.08	0.1

a *: significant P value<0.05*

**Table 3:** *Group C: *Canal enlargement at various levels with ProTaper instruments *(mm)*

**Levels**	**Outer Curvature**		**Inner Curvature**		**P value**
	**Mean**	**SD**	**Mean**	**SD**	
1	0.15	0.08	0.08	0.05	0.08
2	0.19	0.08	0.09	0.04	0.03 a
3	0.20	0.08	0.11	0.04	0.04 a
4	0.19	0.06	0.11	0.04	0.02 a
5	0.16	0.07	0.15	0.05	0.73
6	0.14	0.05	0.22	0.07	0.006 a
7	0.11	0.04	0.24	0.05	0.002 a
8	0.14	0.05	0.24	0.05	0.007 a
9	0.20	0.05	0.26	0.05	0.09
10	0.23	0.09	0.22	0.05	0.1
11	0.22	0.07	0.18	0.05	0.22
12	0.31	0.08	0.16	0.05	0.009 a
13	0.35	0.09	0.21	0.08	0.02 a
14	0.38	0.15	0.28	0.05	0.6

a *: significant P value<0.05*

Each acrylic block was then imaged post-instrumentation as described previously. The image was centered so as to superimpose the pre-instrumentation image over the present image. Three markings made on each acrylic block were used as reference points during superimposition of pre and post-instrumentation images. Pre-instrumentation Images were color coded to differentiate them from the post-instrumentation ones. The enlargement of the canal was calculated based on the amount of resin material removed both from the inner and outer curvature of the canal from the superimposed image at each level.

The enlargement of the canal was calculated based on the amount of resin material removed both from the inner and outer curvature of the canal from the superimposed image at each level. The first measuring point was 0.5 mm from the apical end of the canal, and the final measuring point was 14.5 mm from the apical end. Overall, 14 measuring points were taken at the outer and inner side of the canal. Adobe Photoshop software was used for measuring the distance between pre and post-instrumentation images. Superimposed image on the pre and post-instrumentation image were marked at each level both for both inner and outer curvatures (Figure 1) with the help of calibrating tool in the Adobe Photoshop software. When two points were marked, the difference between the two points was automatically generated by the software and the value obtained was within ±0.01 mm precision. All the measurements were carried out by two blind operators at ×10 magnification. As all the readings were supplied by the software, chances of error were marginalized.

**Table 2:** *Group B:* Canal enlargement at various levels with ProFile instruments (*mm*)

**Levels**	**Outer Curvature **		**Inner Curvature**		**P value**
	**Mean**	**SD**	**Mean**	**SD**	
1	0.09	0.04	0.10	0.00	0.35
2	0.09	0.04	0.10	0.00	0.35
3	0.14	0.07	0.13	0.05	0.73
4	0.15	0.05	0.13	0.05	0.45
5	0.16	0.05	0.13	0.05	0.28
6	0.16	0.05	0.15	0.05	0.68
7	0.16	0.09	0.15	0.05	0.80
8	0.16	0.09	0.19	0.08	0.66
9	0.16	0.09	0.20	0.08	0.52
10	0.23	0.12	0.18	0.12	0.56
11	0.23	0.18	0.20	0.08	0.78
12	0.23	0.18	0.19	0.08	0.67
13	0.25	0.11	0.21	0.08	0.54
14	0.26	0.12	0.18	0.13	0.31

*a: significant P value<0.05*

Results were analyzed using the students ANOVA, Student-Newman-Keuls and paired t-test. Since this study had more than two groups, ANOVA test was chosen for analysis.

## RESULTS

*Group A:* Results (Table 1) demonstrated difference in width between the inner and outer curvature throughout the canal; moreover, at levels 3 and 13 significantly greater amount of resin material was removed from the outer curvature and at levels 6, 7, and 8 significantly greater amounts were removed from the inner curvature.

**Table 4:** Overall comparison of canal enlargement at the outer curvature *(mm)*

**Levels**	**K3**		**Prpfile**		**ProTaper**		**P value**
	**Mean**	**SD**	**Mean**	**SD**	**Mean**	**SD**	
1	0.11	0.06	0.09	0.04	0.15	0.08	0.17
2	0.15	0.11	0.09	0.04	0.19	0.08	0.09
3	0.19	0.08	0.14	0.07	0.20	0.08	0.33
4	0.21	0.08	0.15	0.05	0.19	0.06	0.44
5	0.16	0.05	0.16	0.05	0.16	0.07	0.20
6	0.10	0.05	0.16	0.05	0.14	0.05	0.12
7	0.09	0.04	0.16	0.09	0.11	0.04	0.08
8	0.11	0.04	0.16	0.09	0.14	0.05	0.45
9	0.15	0.05	0.16	0.09	0.20	0.05	0.45
10	0.19	0.06	0.23	0.12	0.23	0.09	0.77
11	0.24	0.07	0.23	0.18	0.22	0.07	0.57
12	0.26	0.11	0.23	0.18	0.31	0.08	0.21
13	0.29	0.08	0.25	0.11	0.35	0.09	0.31
14	0.31	0.21	0.26	0.12	0.38	0.15	0.44

*a: significant P value<0.05*

**Table 5:** Overall comparison of canal enlargement at the inner curvature *(mm)*

**Levels**	**K3 **		**Profile**		**ProTaper**		**P value**
	**Mean**	**SD**	**Mean**	**SD**	**Mean**	**SD**	
1	0.06	0.05	0.10	0.00	0.08	0.05	0.27
2	0.08	0.05	0.10	0.00	0.09	0.04	0.27
3	0.11	0.04	0.13	0.05	0.11	0.04	0.54
4	0.12	0.07	0.13	0.05	0.11	0.04	0.65
5	0.13	0.05	0.13	0.05	0.15	0.05	0.34
6	0.16	0.05	0.15	0.05	0.22	0.07	0.004^a^
7	0.21	0.08	0.15	0.05	0.24	0.05	0.007 a
8	0.22	0.07	0.19	0.08	0.24	0.05	0.33
9	0.19	0.04	0.20	0.08	0.26	0.05	0.005 a
10	0.20	0.05	0.18	0.12	0.22	0.05	0.06
11	0.20	0.05	0.20	0.08	0.18	0.05	0.77
12	0.20	0.09	0.19	0.08	0.16	0.05	0.35
13	0.26	0.08	0.21	0.08	0.21	0.08	0.15
14	0.20	0.08	0.18	0.13	0.28	0.62	0.17

a *: significant P value<0.05*

*Group B:* There was no significant difference between the change in width between the outer and inner curvatures by ProFile files (Table 2) at all 14 levels.

*Group C:* The change in width by ProTaper files (Table 3) were showing significant P values at level 2,3,4,12,13 from the outer and at level 6,7,8 from the inner curvature.

The amount of material removed from the outer curvatures between groups A, B and C was not significantly different (Table 4). However, there was significant difference in the thickness of material removed from the inner curvature of the middle third of the canal between group C (ProTaper) and the other two groups, *i.e. *levels 6, 7 and 9 (Table 5). Group A (K3) and group B (ProFile) rotary instruments did not demonstrate significant difference at these levels.

There were no incidences of ledge formation, fractures or any mishaps during canal preparation.

## DISCUSSION

The use of a simulated canal in a clear resin block allows standardization of the root canal preparation and is an ideal experimental model to allow direct comparison of the shaping ability of different instruments (11). However, a drawback is that resin blocks do not reflect the action of the instruments in root canals of natural teeth because of differences in the surface texture, hardness and cross-section.

When the canal curvature reaches 30 degrees or more, the complexity of the case increases markedly; therefore blocks with 35 degrees root canals curvatures in one plane were chosen for this study (12).

In group A (K3), there is apical transportation at level 3 from the apex. The results were in accordance with the previous studies of Yoshimine *et al.*, Schafer *et al.* and Ayar *et al.* (10,13,14). The reason can be attributed to the modified U-shaped file design of K3 instruments and the three radial land areas that have a positive 45-degree rake angle (15).

There is a significant difference at level 6, 7, and 8 in the inner curvature of the middle third of the canal of group A. It is possible that 0.06 taper or larger taper instruments are stiffer than ISO 0.02 or 0.04 taper, causing inner widening of the middle level of the canal.

Cross section of a ProFile instrument (Group B) shows a U-shape design with radial lands and a parallel central core. Lateral views show a 20-degree helix angle, a constant pitch, and bullet-shaped non cutting tips. Together with a neutral or slightly negative rake angle, this configuration ensures a planning or scraping action on dentin rather than cutting (16). This design feature could be the reason for uniform canal preparation and the absence of significant difference in group B.

The fact that some canal transportation towards the outer aspect of the canal was evident with ProTaper files (Group C) may be because of the variable tapers along the cutting surface of these files. Certainly the decreasing taper sequence of the finishing file enhances the strength of the files, but it increases the stiffness of their tips (17). F1, F2 are finishing files with tip diameters of 0.2 and 0.25 mm respectively. These instruments have a fixed taper of 7% and 8% in the first 3 mm from levels D0 to D3. The larger instruments are stiffer and cause high lateral forces in curved canals (18). These restoring forces attempt to return the file to its original shape and act on the outer side on the canal wall during preparation (17). The significant change in width of the outer curvature at levels 2, 3 and 4 in group C of the present study, may be a result of this.

The ProTaper system has been found to incorporate instruments of progressive multi-taper design with sharp cutting blades. The convex triangular cross section design of the cutting blade is designed to increase the flexibility of the instrument and also increase its cutting efficiency (19). S1 has an increasing taper from 2% on D1 to 11% on D14. S2 has an increasing taper from 4% on D1 to 11.5% on D14. This instrument design could also be one of the reasons for significant change in width from the inner curvature of the middle third of the canal at level 6, 7 and 8. The results of the present study were in accordance to the previous studies of Yun *et al.* and Schafer *et al.* (17,19).

SX shaping file at levels 6, 7, 8 and showed an increase in diameter from 0.5 mm, 0.7 mm, 0.9 mm and 1.1 mm according to taper of 11%, 14.5%, 17% and 19% respectively (8). This could be the reason for significant change in width from the outer curvature at level 12 and 13. 

All the instruments removed more resin material from outer canal curvature in the apical third of the canal and more resin material from the inner canal curvature in the middle third of the canal preparation. This can lead to straightening of the canal in the roots having minimal dentinal thickness.

An overall comparison of the three rotary instruments at the various cross sections illustrated significant different thicknesses removed from the inner curvature of the middle third of the canal at levels 6, 7 and 9 (Table 5). This difference is mainly caused by ProTaper instruments. This may be a consequence of ProTaper variable increasing tapers and sharp triangular cutting edges.

One limitation of this study is the obvious difference in hardness between the resin blocks and dentin. The cutting and shaping ability of rotary instruments will therefore be different. For definitive conclusions, studies on natural teeth are required to corroborate with the findings of the present study.

## CONCLUSION

Progressive taper instrument, ProTaper removed more resin in the apical and middle third of the canal when compared to the constant tapers K3, and ProFile instruments, which did not significantly differ in resin removal in that region. Overall, we can conclude that the instruments maintained original canal curvature relatively well. There was no incidence of ledge formation, fractures or mishaps during canal preparation.
